# The effect of a daily application of a 0.05% chlorhexidine oral rinse solution on the incidence of aspiration pneumonia in nursing home residents: a multicenter study

**DOI:** 10.1186/s12877-017-0519-z

**Published:** 2017-06-19

**Authors:** Vanessa R. Y. Hollaar, Gert-Jan van der Putten, Claar D. van der Maarel-Wierink, Ewald M. Bronkhorst, Bert J. M. de Swart, Nico H. J. Creugers

**Affiliations:** 1 Department of Neurorehabilitation, HAN University of Applied Sciences, P.O. Box 6960, Nijmegen, GL 6503 The Netherlands; 2 BENECOMO, Flemish-Netherlands Geriatric Oral Research Group, Ghent, Nijmegen Belgium; 3 Department of Oral Function and Prosthetic Dentistry, Radboud University Nijmegen Medical Centre, P.O. Box 9101, Nijmegen, HB 6500 The Netherlands; 4 Amaris Gooizicht, Paulus van Loolaan 21, Hilversum, SH 1217 The Netherlands; 5 Center for Special Care in Dentistry, Gustav Mahlerlaan 3004, Amsterdam, LA 1081 The Netherlands; 6 Department of Preventive and Restorative Dentistry, Radboud University Nijmegen Medical Centre, P.O. Box 9101, Nijmegen, HB 6500 The Netherlands; 7 Department of Rehabilitation, Division Speech Therapy, Radboud University Nijmegen Medical Centre, P.O. Box 9101, Nijmegen, HB 6500 The Netherlands

**Keywords:** Oral health care, Oral hygiene care, Chlorhexidine, Pneumonia, Aspiration pneumonia, Dysphagia, Nursing home

## Abstract

**Background:**

Dysphagia and potential respiratory pathogens in the oral biofilm are risk factors for aspiration pneumonia in nursing home residents. The aim of the study was to examine if the daily application of 0.05% chlorhexidine oral rinse solution is effective in reducing the incidence of aspiration pneumonia in nursing home residents with dysphagia. Associations between background variables (age, gender, dysphagia severity, care dependency, medication use, number of medical diagnoses, teeth and dental implants, and wearing removable dentures) and the incidence of aspiration pneumonia were also examined.

**Methods:**

This study is a multicenter study in which for 1 year participants with dysphagia in the intervention group received the usual oral hygiene care with the addition of a 0.05% chlorhexidine oral rinse solution, whereas participants in the control group received only oral hygiene care.

**Results:**

Data of 103 participants in 17 nursing homes were analyzed. Survival analysis showed no significant difference in the incidence of pneumonia between both groups (Cox regression, HR = 0.800; 95% CI [0.368–1.737], *p* = 0.572). Cox regression analysis for Functional Oral Intake Scale (FOIS)-level showed a significant risk of the incidence of pneumonia (HR = 0.804; 95% CI [0.656–0.986], *p* = 0.036). After adjustment for Group and FOIS-level, Cox multivariate proportional hazard regression analysis showed that the variables age, gender, Care-dependency Scale-score (CDS) number of diseases, medication use, number of teeth, and the presence of dental implants or removable dentures were not significantly associated with the incidence of pneumonia.

**Conclusions:**

Chlorhexidine oral rinse solution 0.05% as an adjunctive intervention in daily oral hygiene care was not found to reduce incidence of aspiration pneumonia. The requested number of participants to achieve sufficient power was not established and high drop-out rate and non-structural compliance was present. The power was considered to be sufficient to analyze the associations between the background variables and the incidence of pneumonia in the included nursing home residents with dysphagia. Dysphagia was found to be a risk factor for aspiration pneumonia.

**Trial registration:**

Registration in The Netherlands National Trial Register**:** TC = 3515. Approval for the study was obtained from the Medical Ethical Committee of the Radboud University Medical Center (NL. nr:41,990.091.12).

## Background

In nursing home residents aspiration pneumonia is the second-most-common infection and causes high mortality [1–3]. Pneumonia can be provoked by aspiration of (oral) pathogens into the lower respiratory tract [4, 5]. Aspiration can be induced by dysphagia [6]. Caused by the aging process, significant changes in swallowing occur, which increase elderly peoples risk of dysphagia [7, 8]. Several studies confirm that the presence of dysphagia in nursing home residents is a risk factor for aspiration, which may lead to aspiration pneumonia and, possibly, death [5, 6, 9–14]. The prevalence of dysphagia in nursing homes varies between 38% and 69.6% [15–17] and up to 30% of the elderly with dysphagia develop aspiration [18].

In the oral cavity many structures contain bacteria. These bacteria create a reservoir for pulmonary infections in nursing home residents. Potential respiratory pathogens, such as *Staphylococcus aureus*, *Klebsiella pneumoniae*, *Pseudomonas aerugionosa* and *Enterobacter cloacae*, have been found in the oral biofilm of care-dependent elderly people [4, 19]. Colonization of the oropharynx by these pathogens is an important process in the pathogenesis of aspiration pneumonia in care-dependent elderly people. This is because the normal microflora of their oral biofilm becomes more rapidly colonized by potential pathogens due to diminished salivary secretion rates and a poor ability to perform oral hygiene care. This makes it of great importance to reduce the oral biofilm by carrying out daily oral hygiene care in these populations [20, 21].

It is a challenge to maintain good oral health into old age because in many elderly people functional and cognitive decline results in difficulties with performing daily oral hygiene care [22]. Additionally, nursing staff, including registered nurses and care assistants have a lack of knowledge of and training in, and experience barriers when providing oral hygiene care [23–25]. In contrast, it is known that oral hygiene care and oral hygiene care programs have contributed to preventing nursing home residents from developing pneumonia [12, 20, 21, 26]. It is still unclear which oral hygiene care program or which interventions are most effective in reducing pneumonia.

Mouth rinses that contain chlorhexidine might be helpful in improving oral hygiene care. Chlorhexidine has been found to be effective in decreasing the amount of dental plaque and in reducing certain aerobic and anaerobic species [27–29]. Chlorhexidine has a bactericidal and bacteriostatic activity with a wide spectrum of antibacterial activity, which includes Gram-positive and Gram-negative bacteria [30]. The antibacterial action is explained by the fact that the cationic chlorhexidine molecule is attracted by the negatively charged bacterial cell surface. Then, by penetrating the bacterial cell membrane, leakage of cell components is caused. Disruption of the bacterial metabolism and inhibition of cell growth takes place and finally the bacterial cell dies [30]. In this way, the formation of dental plaque is disturbed.

In critically ill patients, the use of chlorhexidine oral rinse during oral hygiene care has been found to reduce nosocomial pneumonia and ventilator associated pneumonia (VAP) [31]. Furthermore, a systematic review suggested a 0.12% chlorhexidine oral rinse as an effective hygiene method for intensive care unit (ICU) patients in order to reduce nosocomial pneumonia, and the use of chlorhexidine was suggested as more favorable than tooth brushing only [32]. However, because of the high prevalence of dysphagia and aspiration pneumonia and the absence of evidence regarding which oral hygiene care program or which interventions are most effective in reducing pneumonia in frail elderly, more studies are needed to establish an evidence-based oral hygiene care protocol in order to protect nursing home residents from aspiration pneumonia [15–17, 33]. It is unknown whether the daily application of a 0.05% chlorhexidine oral rinse solution is effective in reducing the risk of aspiration pneumonia in nursing home residents.

Against this background, this study was designed to examine, first, whether a daily application of a 0.05% chlorhexidine oral rinse solution in addition to usual daily oral hygiene care is effective in reducing the risk of aspiration pneumonia in physically disabled nursing home residents with dysphagia. The second aim was to examine whether any associations could be found between several background variables (such as age, gender, dysphagia severity, care-dependency score, number of medical diagnoses and medication use, the number of teeth and implants present, and presence of removable dentures) and the risk of acquiring pneumonia in participating physically disabled nursing home residents with dysphagia.

### Primary and secondary outcomes

The primary outcome of the study is the incidence of aspiration pneumonia in physically disabled nursing home residents with dysphagia. Secondary outcomes are possible associations between age, gender, dysphagia severity, care-dependency score, number of medical diagnoses and medication use, the number of teeth and implants present, the presence of removable dentures and the incidence of pneumonia in physically disabled nursing home residents with dysphagia.

## Methods

### Study design and setting

**Fig. 1:**
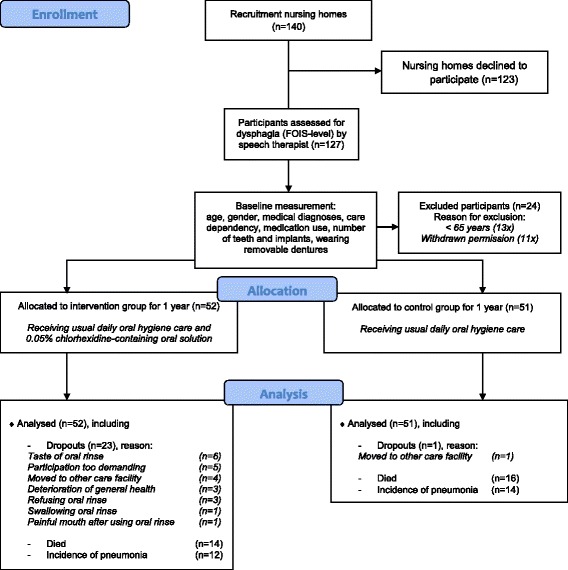
Flow chart of multicenter study

This study design was described and published previously [34] and was developed in accordance with the Consolidated Standards of Reporting Trials (CONSORT) guidelines [35]. The study was conducted in selected nursing homes in The Netherlands (Fig. 1).

The study was originally designed as a multicenter cluster randomized controlled clinical trial, with the nursing home as the unit of randomization [34]. Participation in the intervention group or control group was planned to be assigned at random. However, during the recruitment of the nursing homes, the nursing homes wanted to know in advance to which group their nursing home would be allocated, in order to determine whether they would participate. Arguments given by the nursing homes were the prevention of work overload, introduction of electronic residents’ files, cuts in budget, renovations and merging nursing homes, and upcoming changes in the Dutch health system.

The most important argument for participating given by the respective nursing homes was to improve quality of (oral) health care. All nursing homes emphasized the importance of this study because they were aware of the need for good oral hygiene care in nursing home residents. Therefore, the enrollment of all of the units of these nursing homes took place according to their preference for being in the intervention or control group and this study became a multicenter controlled clinical trial.

### Power calculation

Based on a power calculation, 500 physically-disabled nursing home residents with dysphagia were to be followed during one year (Fig. 1). Calculating the sample size, the incidence of pneumonia in nursing home residents is, in agreement with internationally published incidence data for the year 2011, largely assessed as 25% [36] the presumed reduction of the incidence is 42%, the requested power is 0.8, and the alpha is 0.05. If the true relative risk of failure of residents of the intervention group relative to residents of the control group is 0.58, 225 residents are needed in the intervention group and 225 residents in the control group. It is intended to include 12 nursing home in the study (k = 12) and we assume the ICC to be 0.01. This gives a design effect of 1.11. Taking that into account, the size of both groups needs to be 250.

### Inclusion of participants

Residents were included if they met the following criteria: aged 65 or older, physically disabled, resident in a long-term care unit, and previously diagnosed with dysphagia. In The Netherlands, nursing homes contain somatic and psychogeriatric wards of primarily physically-disabled residents and/or primarily cognitively-impaired residents respectively [37]. Psychogeriatric units, nursing home units with residents for short-term care or with cognitively-impaired residents (mainly suffering from dementia) were excluded from this study. The nursing staff, speech therapist and the responsible elderly-care physician [38] indicated whether the resident was cognitively and physically able to participate and was able to carry out the instructions given by the nursing staff. In addition, the speech therapist of the nursing home determined whether dysphagia was present by using the Functional Oral Intake Scale (FOIS). Dysphagia was assessed as a dysfunction in the process of oral intake by using FOIS to indicate the functional oral intake of food and liquid and the severity of the dysphagia. FOIS has been found to be an adequate reliable and valid instrument for functional oral intake [39]. FOIS is a 7-point scale (levels 1 to 7). Residents with level 1 (nothing by mouth) up to level 6 (total oral diet with multiple consistencies without special preparation but with specific food limitations) were included in the study. Level 7 indicates a total oral diet with no restrictions and therefore no dysphagia [39, 40]. Residents with FOIS-level 7 will not be included. Exclusion criteria for residents were: being cognitively impaired (mainly suffering from dementia), in a coma or vegetative state, terminally ill, dependent on mechanical ventilation, in short-term care, or already using an additional oral hygiene care solution.

During the study, a physician could withdraw a participant from the study when the participant’s condition had altered so that it fitted one of the exclusion criteria. If pneumonia was the reason for the condition alteration, the pneumonia was registered. Withdrawn residents were not replaced because, by replacement, bias would occur in determining the incidence of pneumonia.

### Intervention

The intervention consisted of applying a 0.05% chlorhexidine-containing oral rinse solution (Perio-aid® Maintenance 0.05%, Dentaid BeNeLux BV) twice daily, immediately after the usual oral hygiene care. The application method depended on the severity of the dysphagia. A local speech therapist indicated the safety of the application method by considering the FOIS-level of the resident. Residents who had no problems with thin liquids had to rinse with the 0.05% chlorhexidine-containing solution for 30 s, immediately after the usual oral hygiene care. Residents with severe dysphagia, who could not tolerate thin liquids, had to clean their teeth, gums, tongue, palate, and buccal mucosa with a gauze soaked in a 0.05% chlorhexidine-containing solution, immediately after their usual oral hygiene care.

Participants in the control group received the usual oral hygiene care without the addition of an oral rinse. In both groups, the usual oral hygiene care was not standardized because the introduction of a standard protocol would have been a secondary intervention, which would have disturbed the effect of the chlorhexidine intervention.

### Data collection

In each nursing home, the examination period for the participating residents was 12 months and the examination period for all participants in each nursing home started at the same time. Enrollment of the nursing homes took place from April 2013 to April 2014. This meant that data collection took place from April 2013 to April 2015. During the study period, Case Report Forms (CRFs) were used to collect data regarding the incidence of pneumonia and other medical alternations, such as hospitalization. In both groups, the number of dropouts and deaths and the cause of each dropout or death were registered. Data on any reported adverse effects of the oral rinse were collected from the intervention group.

### Baseline data

After dysphagia (FOIS-levels 1 to 6) had been determined, the other baseline data were collected. Other baseline data on age, gender, actual medical diagnoses and medication use were collected from medical records. If baseline data were incomplete, the resident was excluded and these data were not used for further analysis. The medical diagnoses and conditions and actual medication use were extracted from the residents’ medical files by the principal investigator (first author).

Care Dependency Scale (CDS) was used to determine the degree of care dependence of the resident and is a valid, reliable and cross cultural comparative instrument [41]. At baseline, a caregiver from the nursing staff selected 1 of the 5 care dependency criteria of all 15 items of CDS.. The principal investigator calculated the actual CDS-score. Low scores on the items indicate that patients are completely dependent on care and high scores mean that patients are almost independent of care [41].

The principal investigator and a study assistant performed an oral examination. The oral status was determined by counting the number of teeth, number of implants and number of retained tooth roots. The use of removable dentures or partial prosthesis was also registered.

### Monitoring participants

The participants in the intervention group, who received the application of the chlorhexidine-containing solution daily, were guided and monitored after each period of two weeks by a group of visiting study assistants. During the visits, the study assistants checked the participants on the presence of side effects, the availability of chlorhexidine solution, and possible compliance issues. They also met with an internal study supervisor. After each visit, the study assistants reported their findings on a CRF. In each nursing home, two trained internal study supervisors monitored the usual daily oral hygiene care, including the application of the chlorhexidine-containing solution. The principal investigator visited the intervention groups to evaluate the study with the internal study supervisors after six and twelve months from the start of the study. During those visits, the principal investigator performed an oral examination in the intervention group to monitor potential side effects of the chlorhexidine-containing solution. Study assistants also monitored the participants in the control groups with the same monitoring protocol after each period of four weeks. In the control group, the principal investigator evaluated the ongoing study with the internal study supervisors after six months and at the end of the study.

If symptoms of pneumonia occurred in a participant, they were physically examined by a physician. No established clinical symptoms were available to make a precise clinical distinction between pneumonia and aspiration pneumonia. Therefore, all episodes were reported and counted as pneumonia. If pneumonia was diagnosed, the physician informed the principal investigator in order to register this episode of pneumonia. The diagnosis and related clinical symptoms of pneumonia were registered by the physician. If a participant had more than one episode of pneumonia during the study period of a year, all episodes were monitored and reported.

During the study, where participants were withdrawn from the study by a physician, the physician stated the reason for and date of withdrawal and noted these on a CRF. If the participants wanted to drop out of this study early, their reason and date of dropout were registered on a CRF by a physician or member of the nursing staff.

In both groups, possible adverse events related to study participation were monitored. Signs of objection expressed by the participants or deterioration of their health were closely monitored too. Where any objection was raised or deterioration of participants’ health occurred, the study stopped immediately. Any other alternations in medical conditions of the participants, such as hospitalization, death or discontinuation of the study, were reported by the physician using different CRFs. The study assistants collected all CRFs every two weeks. No follow-up monitoring of the participating nursing home residents took place after the end of the study.

### Ethics and informed consent

The study was conducted according to the principles of the Declaration of Helsinki (version 7c, 2004) and in accordance with the Medical Research Involving Human Subjects ACT (WMO). Approval for the study was obtained from the Medical Ethical Committee of the Radboud University Medical Center (NL.nr: 41,990.091.12). The trial was registered in The Netherlands in the National Trial Register**:** TC = 3515. Written informed consent by the board of each nursing home that participated was received before the start of the study. After this informed consent, a physician and a speech therapist of each nursing home recruited participants from a long-term care unit. The recruited residents or their legal representatives received information about the study, both verbally and in writing. Written informed consent was obtained from all participants or their legal representatives.

### Statistical analysis

The primary outcome variable of this study was an episode of pneumonia in a participating nursing home resident. The effect of the intervention on the occurrence of pneumonia (time to event) was analysed by survival analysis using Cox regression. Survival was defined as the period of time in which a participant did not suffer from pneumonia and/or did not die during the study period. Participants who died were censored at the time of their death. In addition to this main analysis, Cox’s regression model was used to identify risk factors that might influence the incidence of pneumonia. Because the variables group (intervention or control) and FOIS-level determined if and how the chlorhexidine solution was used, these variables were chosen as covariates in the Cox’s regression model to identify other risk factors. This meant that a Cox’s multivariate proportional hazard regression model was built using these two predefined covariates: group (intervention or control) and FOIS-level. The following variables: age, gender, CDS, number of diseases and used medication, number of teeth and the presence of dental implants or removable dentures, were added to the model. Cox’s multivariate proportional hazard regression was used to calculate hazard ratios, confidence intervals and *p*-values. A 95% confidence interval was used with all estimated values. *P*-values were two-tailed and a *P*-value of <0.05 was considered to be statistically significant. Where participants were withdrawn prematurely or dropped out of the study, the moment and reason of withdrawal or dropout were recorded.

## Results

A total of 140 nursing homes in The Netherlands were approached and invited to take part in this study. Seventeen nursing homes agreed to participate: 10 nursing homes agreed to host the intervention group and 7 nursing homes agreed to host the control group. The nursing homes were spread throughout The Netherlands.

At baseline, 127 residents gave their consent to participate in this study. Of these, 13 were not included because it appeared that they had not yet reached the minimal age of 65. During the study, an additional 11 participants were excluded because they withdrew their permission to participate or the baseline data could not be fully collected. Thus, the data of 103 participants were analyzed. These participants were spread over the various nursing homes, with 3 to 7 participants in the intervention group and 2 to 13 participants in the control group per nursing home. The intervention group comprised 52 participants. Following the criteria of the study protocol, speech therapists at the various nursing homes allocated 41 participants (79%) to the ‘rinse-group’ and 11 (21%) to the ‘gauze-group’. The control group comprised 51 participants. Table 1 presents relevant characteristics of the participants, such as age, FOIS-level, CDS-score, number of diseases and medication. Of the participants, 33 (32%) were dentate and 70 (68%) were edentulous. Of the edentulous participants, 65 individuals (93%) wore a complete removable denture while 5 (7%) did not (Table 2).

**Table 1:** Numbers of participants or gender and mean (Mean ± SD) age, FOIS-level, CDS-score, and numbers of diseases and medications in each group at baseline

	Intervention	Control	Total
Number of participants	52	51	103
Number of men / women	25/27	26/25	51/52
Age	79.4 ± 8.9	81.7 ± 9.03	80.5 ± 9.0
FOIS-level	4.8 ± 1.5	4.9 ± 1.5	4.8 ± 1.5
CDS-score	38.0 ± 16.6	35.0 ± 15.0	36.5 ± 15.8
Number of diseases	4.7 ± 2.4	4.3 ± 2.2	4.5 ± 2.3
Number of medication	8.6 ± 3.7	9.6 ± 3.6	9.0 ± 3.7

**Table 2:** Number of participants with a certain oral status determined by their oral examination at baseline

	Intervention	Control	Total
Dentulous, including participants with	14	19	33 (32%)
Natural teeth	6	11	17
Dental implant(s)	4	1	5
Partial prosthesis	1	1	2
Removable denture	3	4	7
Removable denture and partial prosthesis	0	2	2
Mean number of teeth	15.1 ± 7.9	14.6 ± 7.1	14.9 ± 7.3
Edentulous, including participants with	38	32	70 (68%)
Complete removable dentures	29	30	59
Complete removable dentures with dental implants	5	1	6
Not wearing dentures	4	1	5

Forty-eight percent of all participants (*n* = 49) completed the study and could be monitored for the whole study period (Fig. 1 & Table 3). Of these participants, 15 (29%) were assigned to the intervention group and 34 (67%) to the control group. During the study, 24 participants (23%) dropped out: 23 (44%) in the intervention group and 1 (2%) in the control group. Reasons for dropping out were the taste of the oral rinse (*n* = 6), participation was too demanding for the resident (*n* = 5), participant moved to another long-term care facility (*n* = 4), deterioration of general health (*n* = 3), refusing oral rinse (*n* = 3), swallowing oral rinse (*n* = 1), and painful mouth after use of oral rinse (*n* = 1). The participant in the control group dropped out because this participant moved to another long-term care facility. A total of 30 (29%) participants died due to other causes than pneumonia during the study period: 14 in the intervention group and 16 in the control group.

**Table 3:** Number and percentages (%) of participants who participated for one year, dropped out or died during the study period and incidence of pneumonia, including dropouts and mortality in each group

	Intervention *N* = 52	Control *N* = 51	Total *N* = 103
Included 1 year	15 (29)	34 (67)	49 (48)
Dropout	23 (44)	1 (2)	24 (23)
Mortality	14 (27)	16 (31)	30 (29)
Pneumonia	12 (23)	14 (27)	26 (25)
Included 1 year	3 (25)	7 (50)	10 (38)
Dropout	1 (8)	0	1 (4)
Mortality	8 (67)	7 (50)	15(58)

No side effects of the use of a chlorhexidine oral rinse, such as tooth, tongue or denture discoloration, prolonged taste loss or candidosis, were reported by the participants or by the study assistants during their monitoring visits.

**Fig. 2:**
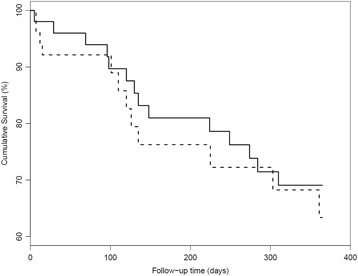
Pneumonia free survival by group status. Legends: Intervention - - - -. Control ─

Pneumonia was diagnosed in 26 (25%) participants: 12 in the intervention group (23%) and 14 in the control group (27%). Of the participants with pneumonia, 15 died (58%) as a result of pneumonia: 8 (67%) participants in the intervention group and 7 (50%) in the control group. A Kaplan-Meier method was used to calculate the survival curves in both groups (Fig. 2). Survival curves in the intervention group showed no significant difference in the incidence of pneumonia compared with the control group (log rank, *p* = 0.571). Cox regression analysis for FOIS-level showed a significant risk of the incidence of pneumonia (HR = 0.804, [95% CI 0.656–0.986], *p* = 0.036) (Table 4). After adjustment for Group and FOIS-level, Cox multivariate regression analysis showed that the variables age, gender, CDS-score, number of diagnoses, medication use, number of teeth, and the presence of dental implants or removable dentures were not significantly associated with the incidence of pneumonia.

**Table 4:** Results of Cox regression analysis for group and FOIS-level of the risk of the incidence of pneumonia and Cox’s multivariate proportional hazards regression model of possible risk factors for pneumonia after correction for group and FOIS-level presented as hazard ratio (HR), 95% confidence interval (95% CI) and *P*-value

	HR	95% CI	*P*-value
Group (Intervention / Control)	0.800^a^	[0.368–1.737]	0.572
FOIS-level	0.804^a^	[0.656 – 0.986]	0.036
Age	0.990	[0.943–1.039]	0.684
Gender	1.017	[0.469–2.205]	0.965
CDS-score	0.976	[0.948–1.006]	0.118
Number of diseases	1.046	[0.873–1.253]	0.628
Number of used medication	1.087	[0.977–1.210]	0.127
Oral status (edentulous/dentate)	1.526	[0.702–3.319]	0.286
Number of teeth	1.002	[0.923–1.089]	0.956
Dental implants (yes/no)	1.104	[0.247–4.941]	0.897
Removable denture (yes/no)	1.057	[0.236–4.725]	0.942

^a^HR-ranges for Group from 0.664 to 0.939 and HR-ranges for FOIS-level from 0.738 to 0.920 in the nine different Cox’s multivariate proportional hazards regression models with possible risk factors

## Discussion

Unfortunately, we were unable to recruit the required number of participants to achieve sufficient power to compare the risk of aspiration pneumonia in nursing home residents with dysphagia who received a daily application of a 0.05% chlorhexidine oral rinse solution in addition to usual daily oral hygiene with those who did not received this intervention. This power issue might explain why we did not find a significant effect from the intervention. However, with regard to the secondary outcome, we consider the power to be sufficient to analyse the associations between the background variables and the incidence of pneumonia in the included nursing home residents with dysphagia.

In addition to that, the percentage of dropouts (44%) and the small percentage of participants left, who were enrolled in the intervention group for a full year (29%), were not able to demonstrate a possible beneficial effect of the chlorhexidine oral rinse on the reduction of aspiration pneumonia. The participants in this study were at greater risk of dying, because they suffered from multimorbidity and polypharmacy (respectively, mean number of diseases 4.5 ± 2.3, number of medication use 9.0 ± 3.7) and were in extremis care dependent (mean CDS-score 36.5 ± 15.8) [42]. The dropout rate in this study seems high, but this dropout rate and reasons for dropping out in the present study are comparable to other studies [23, 43]. Strengths of this study were the long duration, the number of nursing homes spread over The Netherlands, and supervised enrollment and monitoring of the intervention by a study assistant and a staff member. To our knowledge, this is the first reported trial to study the effect of a 0.05% chlorhexidine oral rinse solution on reducing pneumonia in nursing home residents with dysphagia.

The present study could not demonstrate that the daily use of 0.05% chlorhexidine oral rinse solution in addition to the usual oral hygiene care reduced the incidence of aspiration pneumonia significantly, compared to nursing home residents who received only the usual oral hygiene care. Juthani-Mehta et al. (2015) found a similar result among 834 nursing home residents with impaired swallowing function and/or oral hygiene care, who received manual tooth brushing plus a 0.12% chlorhexidine oral rinse, twice per day, together with upright positioning during feeding during in a 2.5 year follow-up study [33]. That study also did not find a reduced incidence of pneumonia, although it was reported that the compliance of the nursing staff with the intervention protocol may have been inadequate for preventing pneumonia. In our study, extensive efforts were made to enhance the compliance by intensive monitoring of the participants in the intervention group and their nursing staff. However, despite the supervised enrollment of participants in the intervention by members of the nursing staff and study assistants, it is unknown in what manner the instructed information was passed from the study team to the nursing staff. In addition, another presumption of non-structural compliance with the protocol was made, arising from the fact that it appeared that less than the calculated amount of oral rinse was used for the entire study. A previous study reported that supervised implementation of an oral health care protocol improves the knowledge of the nursing staff about oral hygiene care, but not their attitude [44]. We were unable to obtain adequate information about the attitude of the nursing staff during this study.

In general, integration of oral hygiene care into daily care in nursing homes is still a problem [45]. It is important to note that a recently published meta-analysis found there is evidence that oral care interventions made by nursing staff probably result in little or no difference in mortality from pneumonia compared to usual oral care. In contrast, oral care interventions made by dental professionals may reduce mortality from pneumonia [46]. These findings strengthen the importance of the contribution made by the expertise and practice of dental hygienists or dentists in nursing homes to providing oral hygiene care and solving oral care challenges in these complex health care settings [47].

In this study a low-concentration and non-alcoholic chlorhexidine oral rinse solution was used to reduce possible side effects of long-term use of chlorhexidine [48]. It is unclear whether a higher concentration of chlorhexidine oral rinse solution or using any (safe) chlorhexidine solution is effective to prevent nursing home residents from developing aspiration pneumonia [33, 49].

A systematic review reported rather strong evidence that oral hygiene care, including chlorhexidine rinse or gel, significantly reduces the risk of VAP. Nonetheless, this review found no evidence that oral hygiene care with chlorhexidine makes a difference to the number of patients who die in ICU; only limited evidence was found on the effect of tooth brushing on the risk of developing VAP [50]. The differences in the reduction of pneumonia found in both studies might be explained by the fact that most ICU patients receive nasogastric tube feeding, which means that there is less aspiration of food into the lower tract. In our study the participating nursing home residents had dysphagia and they mostly received an oral diet, whether or not in multiple consistencies or with specific food limitations. With this in mind, it seems reasonable that ICU patients should be better prevented from aspiration pneumonia than nursing home residents.

This assumption is supported by one of the findings from the secondary outcomes. Dysphagia was found in this study to be a significant risk factor in the development of pneumonia. Dysphagia determined by FOIS-level reflects a dysfunction in the process of oral intake, in which the FOIS-level indicates the functional oral intake of food and liquid and the severity of the dysphagia. No difference in mean FOIS-level was found between the participants in the intervention and control groups, which had respectively mean FOIS-levels of 4.8 (±1.5) and 4.9 (±1.5) (*p* = 0.706). A total mean FOIS-level of 4.8 (±1.5) indicates that all participants had adjustments in their oral diet, mainly in the consistency of their food. These different consistencies of their food vary in order to prevent aspiration. In spite of these adjustments in the oral diets of the residents, they were still at a higher risk of aspiration. This finding confirms that nursing home residents with dysphagia are at a higher risk of pneumonia, which has been shown by previous studies [12, 14, 51, 52].

This finding suggests that nursing home residents should receive more guidance in using strategies to prevent aspiration during eating and drinking, such as not eating or drinking when feeling rushed or tired, eliminating distractions during swallowing, avoiding consuming solid and liquid food at the same time, and using a teaspoon for putting small amounts of food or liquid into the mouth [53]. Tongue cleaning too seems to improve the coughing ability in nursing home residents, which may prevent aspiration [54].

From the findings of this study, it appears that oral intake of food plays a more important role in the onset of aspiration pneumonia than the amount of dental plaque because it seems likely that the oral bacteria are transported by the food into the lungs. After aspiration, it is unclear whether the oral bacteria or the food provoke the eventual pneumonia, as both are possible causes. Future studies are needed to investigate this.

Other factors, such as age, care dependence, and number of diseases or medications, were not established as risk factors for pneumonia in this study. The combination of chronic diseases and geriatric syndromes has a substantial effect on the functional status of the elderly and increases their frailty [55]. It is proposed that the cumulative effect of multimorbidity, amount of medication and care dependence influences the prevalence and degree of frailty and makes nursing home residents more vulnerable to pneumonia. Owing to multiple factors, such as frailty, impaired efficacy of swallowing, decreased cough reflex and neurological complications, dysphagia can also be considered as a geriatric syndrome [56]. The standard performance of a swallowing assessment in all nursing home residents at admission, followed by a regular update and evaluation of their swallowing status, can be helpful for identifying dysphagia.

Based on the findings of this study and the reasons why participants dropped-out, further definitive trial with a 0.05% chlorhexidine oral rinse may not be appropriate. More studies are needed to investigate which interventions are effective in order to establish an evidence-based oral hygiene care protocol to prevent nursing home residents from developing aspiration pneumonia.

## Conclusion

Chlorhexidine oral rinse solution 0.05% as an adjunctive intervention to daily oral hygiene care was not found to reduce the incidence of aspiration pneumonia. The requested number of participants to achieve sufficient power was not established and high drop-out rate and non-structural compliance was present. The power issue might be an explanation why a significant effect from the intervention was not found. With regard to the secondary outcome, the power was considered to be sufficient to analyze the associations between the background variables and the incidence of pneumonia in the included nursing home residents with dysphagia. Dysphagia was found to be a risk factor for aspiration pneumonia and can be considered a geriatric syndrome.

## Abbreviations

CDS: Care dependency scale
CI: Confidence interval
CRF: Clinical report form
FOIS: Functional oral intake scale
HR: Hazard ratio
ICU: Intensive care unit
VAP: Ventilator associated pneumonia

## References

[1] Mylotte JM, Goodnough S, Naughton BJ (2003). Pneumonia versus aspiration pneumonitis in nursing home residents: diagnosis and management. J Am Geriatr Soc.

[2] Shariatzadeh MR, Huang JQ, Marrie TJ (2006). Differences in the features of aspiration pneumonia according to site of acquisition: community or continuing care facility. J Am Geriatr Soc.

[3] Nakagawa N, Saito Y, Sasaki M, Tsuda Y, Mochizuki H, Takahashi H (2014). Comparison of clinical profile in elderly patients with nursing and healthcare-associated pneumonia, and those with community-acquired pneumonia. Geriatr Gerontol Int.

[4] Russell SL, Boylan RJ, Kaslick RS, Scannapieco FA, Katz RV (1999). Respiratory pathogen colonization of the dental plaque of institutionalized elders. Spec Care Dentist.

[5] Ortega O, Sakwinska O, Combremont S (2015). High prevalence of colonization of oral cavity by respiratory pathogens in frail older patients with oropharyngeal dysphagia. Neurogastroenterol Motil.

[6] Marik PE, Kaplan D (2003). Aspiration pneumonia and dysphagia in the elderly. Chest J.

[7] Daggett A, Logemann J, Rademaker A, Pauloski B (2006). Laryngeal penetration during deglutition in normal subjects of various ages. Dysphagia.

[8] Aslam M, Vaezi MF (2013). Dysphagia in the elderly. Gastroenterology & Hepatology.

[9] Langmore SE, Skarupski KA, Park PS, Fries BE (2011). Predictors of aspiration pneumonia in nursing home residents. J Am Med Dir Assoc.

[10] Janssens JP (2005). Pneumonia in the elderly (geriatric) population. Curr Opin Pulm Med.

[11] Eisenstadt SE (2010). Dysphagia and aspiration pneumonia in older adults. J Am Acad Nurse Pract.

[12] El-Solh AA, Niederman MS, Drinka P (2010). Nursing home-acquired pneumonia: a review of risk factors and therapeutic approaches. Curr Med Res Opin.

[13] Van der Maarel-Wierink CD, Vanobbergen JN, Bronkhorst EM, Schols JM, De Baat C (2011). Risk factors for aspiration pneumonia in frail older people: a systematic literature review. J Am Med Dir Assoc.

[14] Van der Maarel-Wierink CD, Vanobbergen JN, Bronkhorst EM, Schols JM, De Baat C (2013). Oral health care and aspiration pneumonia in frail older people: a systematic literature review. Gerodontology.

[15] Nogueira D, Reis E (2013). Swallowing disorders in nursing home residents: how can the problem be explained?. Clin Interv Aging.

[16] Park YH, Han HR, Oh BM (2013). Prevalence and associated factors of dysphagia in nursing home residents. Geriatr Nurs.

[17] Sarabia-Cobo CM, Pérez V, De Lorena P (2016). The incidence and prognostic implications of dysphagia in elderly patients institutionalized: a multicenter study in Spain. Appl Nurs Res.

[18] Rofes L, Arreola V, Almirall J, et al. Diagnosis and management of oropharyngeal dysphagia and its nutritional and respiratory complications in the elderly. Gastroenterol Res Pract. 2011; pii: 818979. 10.1155/2011/818979 PMC2929516 20811545

[19] Sumi Y, Miura H, Michiwaki Y, Nagaosa S, Nagaya M (2007). Colonization of dental plaque by respiratory pathogens in dependent elderly. Arch Gerontol Geriatr.

[20] Yoneyama T, Yoshida M, Ohrui T (2002). Oral care reduces pneumonia in older patients in nursing homes. J Am Geriatr Soc.

[21] Adachi M, Ishihara K, Abe S, Okuda K (2007). Professional oral health care by dental hygienists reduced respiratory infections in elderly persons requiring nursing care. Int J Dent Hyg.

[22] Batchelor P (2015). The changing epidemiology of oral diseases in the elderly, their growing importance for care and how they can be managed. Age Ageing.

[23] De Visschere L, Schols J, Van der Putten GJ, De Baat C, Vanobbergen J (2012). Effect evaluation of a supervised versus non-supervised implementation of an oral health care guideline in nursing homes: a cluster randomised controlled clinical trial. Gerodontology.

[24] Lindqvist L, Seleskog B, Wårdh I, Von Bültzingslöwen I (2013). Oral care perspectives of professionals in nursing homes for the elderly. Int J Dent Hyg.

[25] Wårdh I, Jonsson M, Wikström M (2012). Attitudes to and knowledge about oral health care among nursing home personnel--an area in need of improvement. Gerodontology.

[26] Pace CC, McCullough GH (2010). The association between oral microorganisms and aspiration pneumonia in the institutionalized elderly: review and recommendations. Dysphagia.

[27] Quirynen M, Soers C, Desnyder M, Dekeyser C, Pauwels M, van Steenberghe D (2005). A 0.05% cetyl pyridinium chloride 0.05% chlorhexidine mouth rinse during maintenance phase after initial periodontal therapy. J Clin Periodontol.

[28] Van Strydonck DA, Slot DE, Van der Velden U, Van der Weijden F (2012). Effect of a chlorhexidine mouthrinse on plaque, gingival inflammation and staining in gingivitis patients: a systematic review. J Clin Periodontol.

[29] Prasad M, Patthi B, Singla A, Gupta R, Jankiram C, Kumar JK (2016). The clinical effectiveness of post-brushing rinsing in reducing plaque and gingivitis: a systematic review. J Clin Diagn Res.

[30] Denton GW. Chlorhexidine. In: Block, S.S. (ed.). Disinfections, Sterilization and Preservation, 199;,4:274–289. Philadelphia: Lea and Febiger.

[31] Silvestri L, Weir I, Gregori D (2014). Effectiveness of oral chlorhexidine on nosocomial pneumonia, causative micro-organisms and mortality in critically ill patients: a systematic review and meta-analysis. Minerva Anestesiol.

[32] Vilela MC, Ferreira GZ, Santos PS, Rezende NP (2015). Oral care and nosocomial pneumonia: a systematic review. Einstein (Sao Paulo).

[33] Juthani-Mehta M, Van Ness PH, McGloin J (2015). A cluster-randomized controlled trial of a multicomponent intervention protocol for pneumonia prevention among nursing home elders. Clin Infect Dis.

[34] Hollaar V, Van der Maarel-Wierink CD, Van der Putten GJ, De Swart B, De Baat C. Effect of daily application of a 0.05% chlorhexidine solution on the incidence of (aspiration) pneumonia in care home residents: design of a multicentre cluster randomized controlled clinical trial. BMJ Open. 2015;5:e007889. 10.1136/bmjopen-2015-007889 PMC4710816 26715476

[35] Schulz KF, Altman DG, Moher D, CONSORT Group. CONSORT 2010 Statement: updated guidelines for reporting parallel group randomised trials. BMC Med. 2010;11:32. PMC3116666 21686296

[36] Kerpiçlik F, Haenen A, Alblas J (2012). Surveillance Netwerk Infectieziekten Verpleeghuizen (SNIV). Referentiecijfers basis surveillance 2011.

[37] Schols JM (2005). Nursing home medicine in The Netherlands. Eur J Gen Pract.

[38] Koopmans RT, Lavrijsen JC, Hoek JF, Went PD, Schols JM (2010). Dutch elderly care physician: a new generation of nursing home physician specialists. J Am Geriatr Soc.

[39] Crary MA, Carnaby Mann GD, Groher ME (2005). Initial psychometric assessment of a functional oral intake scale for dysphagia in stroke patients. Arch Phys Med Rehabil.

[40] Hansen T, Kjaersgaard A, Faber J (2011). Measuring elderly dysphagic patients' performance in eating-a review. Disabil Rehabil.

[41] Dijkstra A, Yönt GH, Korhan EA (2012). The care dependency scale for measuring basic human needs: an international comparison. J Adv Nurs.

[42] Caljouw MA, Cools HJ, Gussekloo J (2014). Natural course of care dependency in residents of long-term care facilities: prospective follow-up study. BMC Geriatr.

[43] Van der Putten GJ, Mulder J, de Baat C, De Visschere LM, Vanobbergen JN, Schols JM (2013). Effectiveness of supervised implementation of an oral health care guideline in care homes; a single-blinded cluster randomized controlled trial. Clin Oral Investig.

[44] Janssens B, De Visschere L, Van der Putten GJ, De Lugt-Lustig K, Schols JM, Vanobbergen J (2016). Effect of an oral healthcare protocol in nursing homes on care staffs' knowledge and attitude towards oral health care: a cluster-randomised controlled trial. Gerodontology.

[45] De Visschere L, De Baat C, De Meyer L (2015). The integration of oral health care into day-to-day care in nursing homes: a qualitative study. Gerodontology.

[46] Sjögren P, Wårdh I, Zimmerman M, Almståhl A, Wikström M (2016). Oral care and mortality in older adults with pneumonia in hospitals or nursing homes: systematic review and meta-analysis. J Am Geriatr Soc.

[47] Barnes CM (2014). Dental hygiene intervention to prevent nosocomial pneumonias. J Evid Based Dent Pract.

[48] Todkar R, Sheikh S, Byakod G, Muglikar S (2012). Efficacy of chlorhexidine mouthrinses with and without alcohol – a clinical study. Oral Health Prev Dent.

[49] El-Rabbany M, Zaghlol N, Bhandari M, Azarpazhooh A (2015). Prophylactic oral health procedures to prevent hospital-acquired and ventilator-associated pneumonia: a systematic review. Int J Nurs Stud.

[50] Hua F, Xie H, Worthington HV, Furness S, Zhang Q, Li C (2016). Oral hygiene care for critically ill patients to prevent ventilator-associated pneumonia. Cochrane Database Syst Rev.

[51] Wójkowska-Mach J, Gryglewska B, Romaniszyn D (2013). Age and other risk factors of pneumonia among residents of polish long-term care facilities. Int J Infect Dis.

[52] Rommel N, Hamdy S (2016). Oropharyngeal dysphagia: manifestations and diagnosis. Nat Rev Gastroenterol Hepatol.

[53] Ney DM, Weiss JM, Kind AJH, Robbins J (2009). Senescent swallowing: impact, strategies, and interventions. Nutr Clin Pract.

[54] Izumi M, Takeuchi K, Ganaha S, Akifusa S, Yamashita Y (2016). Effects of oral care with tongue cleaning on coughing ability in geriatric care facilities: a randomized controlled trial. J Oral Rehabil.

[55] Lee PG, Cigolle C, Blaum C (2009). The co-occurrence of chronic diseases and geriatric syndromes: the health and retirement study. J Am Geriatr Soc.

[56] Baijens LW, Clavé P, Cras P (2016). European Society for Swallowing Disorders – European Union geriatric medicine society white paper: oropharyngeal dysphagia as a geriatric syndrome. Clin Interv Aging.

